# Supplementary material for: Prokaryotic communities from a lava tube cave in La Palma Island (Spain) are involved in the biogeochemical cycle of major elements
Source: PeerJ. 2021 May 11;9:e11386. doi: 10.7717/peerj.11386 (PMC8121065; doi:10.7717/peerj.11386)
Supplement: Supplemental Information 1 [file peerj-09-11386-s001.pdf]

Supplemental file

Prokaryotic communities from a lava tube cave in La Palma Island (Spain) are involved in the biogeochemical cycle of major elements

Jose Luis Gonzalez-Pimentel[1], Tamara Martin-Pozas[2], Valme Jurado[3], Ana Zelia Miller[1], Ana Teresa Caldeira[1], Octavio Fernandez-Lorenzo[4], Sergio Sanchez-Moral[2] and Cesareo Saiz-Jimenez[3]

[1]Laboratorio HERCULES, Universidade de Evora, 7000-809 Evora, Portugal
[2]Museo Nacional de Ciencias Naturales (MNCN-CSIC), 28006 Madrid, Spain
[3]Instituto de Recursos Naturales y Agrobiologia (IRNAS-CSIC), 41012 Sevilla, Spain
[4]Grupo de Espeleologia Tebexcorade, La Palma, Spain

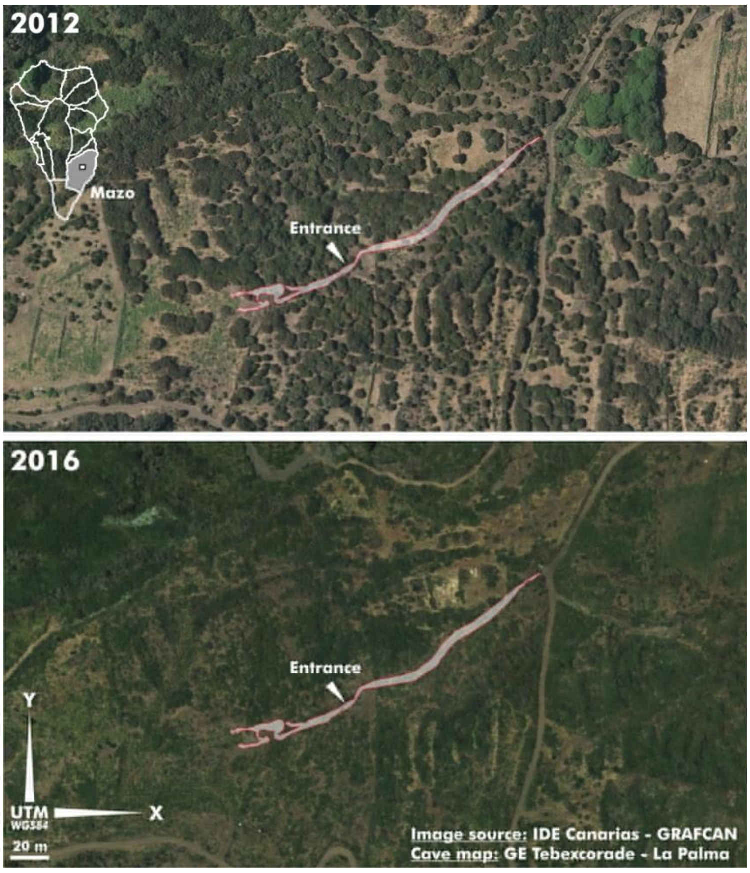

Supplemental Figure 1. Aerial photos of the cave area before the August 4, 2012 fire, and in 2016.

Fuente de la Canaria Cave opens its entrance on a midland slope facing east-southeast, which shows terraces dedicated to rainfed crops, today abandoned, and totally or partially occupied by forage plants, mainly *Chamaecytisus prolifer* subsp. *prolifer* var. *palmensis*, known locally as tagasaste palmero. The vegetation of the area was the evergreen laurel cloud forest with abundance of the wax

myrtle *Myrica faya* and the shrubby *Erica arborea* (tree heath), community known as monteverde or fayal-brezal. The entire area was devastated by a fire in 2012 affecting the surface vegetation and most heaths have been reduced to stumps that, in some cases, are sprouting again from the roots (Supplementary Fig. S1). There are still some specimens in the vicinity of the cave, particularly *Erica arborea*, a species of which one specimen is growing at the cave entrance. Tagasastes are currently the most abundant species. Small herds of cows occasionally graze on the soil above the cave.

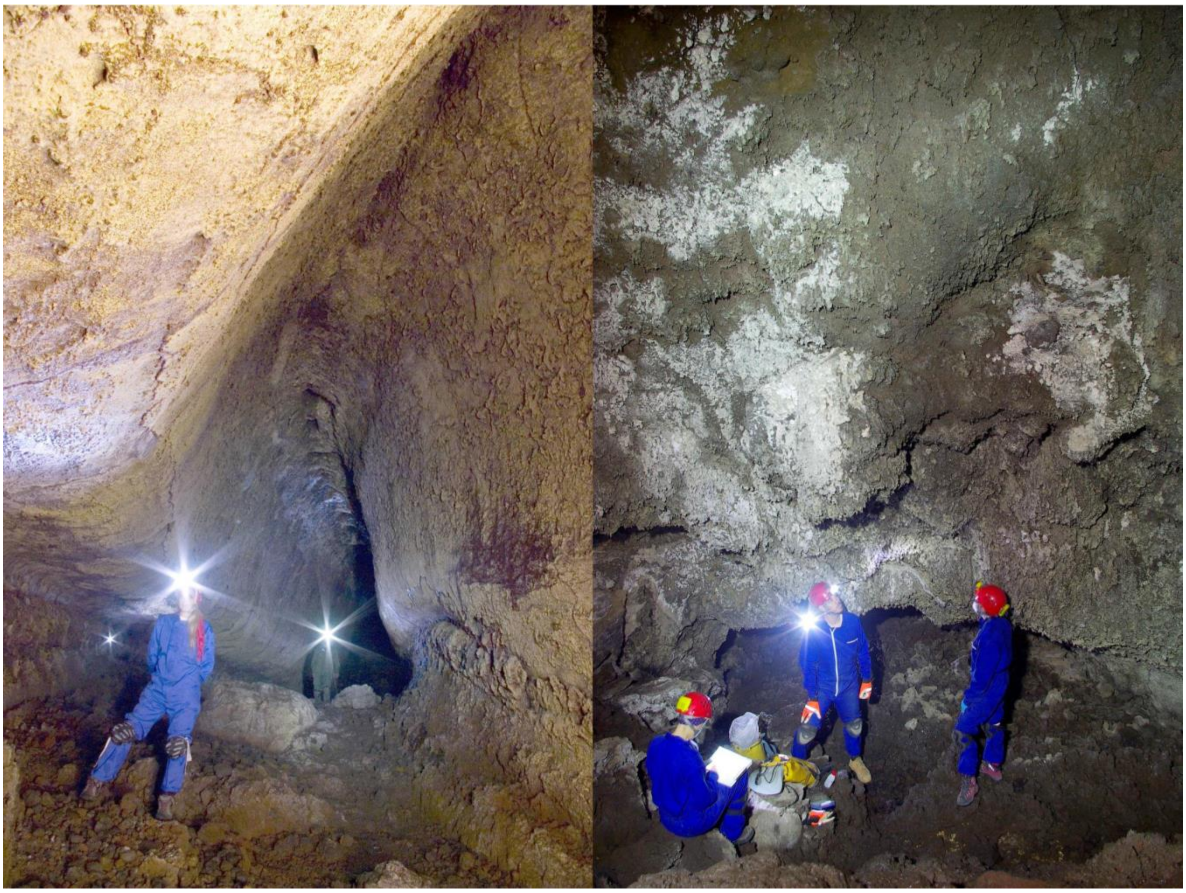

Supplemental Figure 2. Sampling in Fuente de la Canaria Cave. White stains on the walls correspond to the area of moonmilk (MZ03-10J).

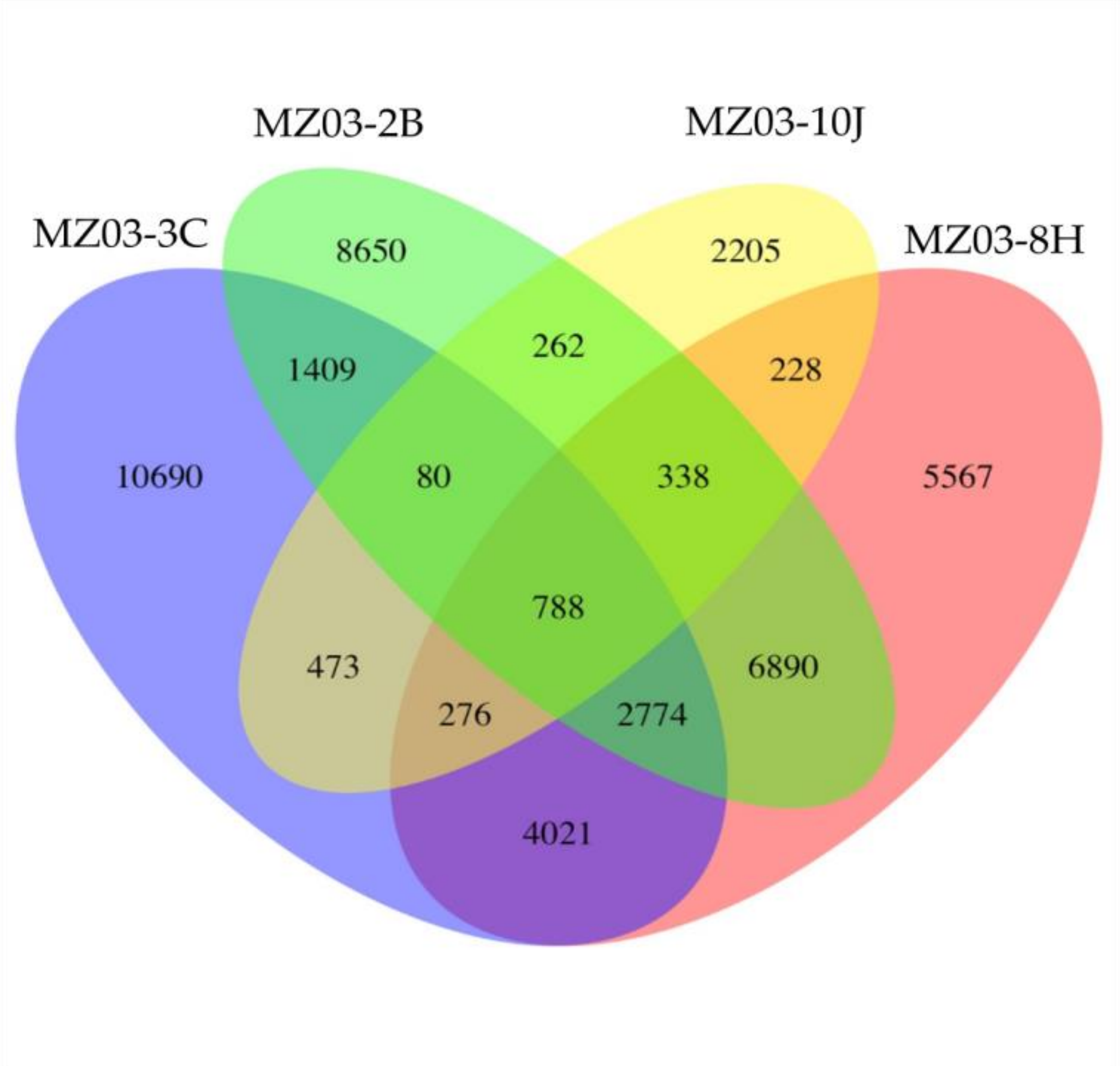

Supplemental Figure 3. Venn diagram at a distance of 0.03 of the shared OTUs among samples MZ03-3C (20511), MZ03-2B (21191), MZ03-8H (20882) and MZ03-10J (4650).

Supplemental Table 1. Selection of enzymes related to methane metabolism. The EC number abundance predicted by PICRUSt2 show how each dominant taxon is contributing to each predicted enzyme per sample.

EC Number	Enzyme description	Taxa	Abundance			
			MZ03-3C	MZ03-8H	MZ03-2B	MZ03-10J
EC:4.2.1.147	5,6,7,8-tetrahydromethanopterin hydro-lyase	Uncultured, *Beijerinckiaceae*	16091	10177	5342	149
		Polaromonas	0	4	3	0
		Hyphomicrobium	3042	3377	1769	773
		Nitrospira	0	0	1	0
EC:1.12.98.1	Coenzyme F420 hydrogenase	Ga0077536	71422	28621	9122	48867
		IMCC26256	1118	7513	4362	0
		Uncultured, *Acidimicrobiia*	1764	5435	2897	0
EC:2.3.1.101	Formylmethanofuran--tetrahydromethanopterin N-formyltransferase	Uncultured, *Beijerinckiaceae*	16091	10177	5342	149
		Polaromonas	0	4	3	0
		Hyphomicrobium	3034	3384	1769	769
		Nitrospira	0	0	1	0
EC:1.14.18.3	Methane monooxygenase (particulate)	Uncultured, *Beijerinckiaceae*	16091	10177	5342	149

		Hyphomicrobium	24	8	1	5
		Nitrospira	6	9	5	0
		MND1	12	432	259	1
		IS-44	2437	3783	2690	43
EC:1.1.2.7	Methanol dehydrogenase	Uncultured, *Beijerinckiaceae*	32182	20354	10684	298
		Hyphomicrobium	6024	6756	3566	1566
		IS-44	4	0	0	0
EC:3.5.4.27	Methenyltetrahydromethanopterin cyclohydrolase	Uncultured, *Beijerinckiaceae*	16091	10177	5342	149
		Nitrospira	0	0	1	0
		Polaromonas	0	4	3	0
		Hyphomicrobium	3034	3384	1769	769
EC:1.4.9.1	Methylamine dehydrogenase	*Hyphomicrobium*	5532	4384	1930	512
		Polaromonas	0	3	18	0
		MND1	1	22	24	1
		IS-44	0	1	3	2

EC:1.5.8.2	Trimethylamine dehydrogenase	*Hyphomicrobium*	4749	2883	1333	2061
EC:1.14.13.148	Trimethylamine monooxygenase	*Hyphomicrobium*	154	1146	753	510

Supplemental Table 2. Selection of enzymes related to sulfur metabolism. The EC number abundance predicted by PICRUSt2 show how each dominant taxon is contributing to each predicted enzyme per sample.

**EC Number**	**Enzyme description**	**Taxa**	**Abundance**			
			**MZ03-3C**	**MZ03-8H**	**MZ03-2B**	**MZ03-10J**
EC:2.7.1.25	adenylyl-sulfate kinase	wb1-P19	47872	17264	5766	299
		Uncultured, *Beijerinckiaceae*	16091	10177	5342	149
		Acidobacteria Subdivision 2	943	4429	3696	1
		PLTA13	6736	1416	79	8
		IMCC26256	1118	7513	4362	0
		Ga0077536	71548	28635	9127	51710
		Nitrospira	17929	18973	13938	1513
		Uncultured, *Euzebyaceae*	9567	2032	7	0
		Uncultured, *Acidimicrobiia*	943	4429	3696	1
		Crossiella	32800	12958	5205	1450

		Elev-16S-976 (*Actinobacteria*)	1624	3011	3365	19
		Hyphomicrobium	2809	4134	2226	1046
		IS-44	1	1	0	0
EC:2.7.7.4	sulfate adenylyltransferase.	wb1-P19	95744	34528	11532	598
		Uncultured, *Beijerinckiaceae*	64364	40708	21368	596
		Acidobacteria Subdivision 2	1886	8858	7392	2
		PLTA13	21072	4973	422	18
		Steroidobacter	1074	253	43	5032
		IMCC26256	2236	15026	8724	0
		Ga0077536	143393	57283	18263	104386
		Uncultured, *Euzebyaceae*	28701	6096	21	0
		11-24 (*Blastocatellia*)	6553	3443	1372	182
		Crossiella	81987	32392	13012	2900
		Candidatus Methylomirabilis	2667	14959	10376	12
		wb1-A12	815	5563	3933	20
		Elev-16S-976 (*Actinobacteria*)	4060	7527	8412	47

		Uncultured, *Burkholderiaceae*	18388	10406	4478	4
		Hyphomicrobium	5428	7182	3734	1537
		Polaromonas	78	11904	7842	58
		MND1	0	5	1	2
		IS-44	58	6054	4322	0
		Nitrospira	16612	19707	14954	1343
EC:1.8.4.8	phosphoadenylyl-sulfate reductase	wb1-P19	47872	17264	5766	299
		Uncultured, *Beijerinckiaceae*	16091	10177	5342	149
		Acidobacteria Subdivision 2	943	4429	3696	1
		PLTA13	14336	3557	343	10
		Steroidobacter	1304	296	53	6855
		IMCC26256	1118	7513	4362	0
		Ga0077536	423	27	14	3809
		Uncultured, *Euzebyaceae*	9567	2032	7	0
		Crossiella	16414	6481	2602	1449
		Elev-16S-976 (*Actinobacteria*)	812	1505	1682	9

		Uncultured, *Burkholderiaceae*	9194	5203	2239	2
		Uncultured, *Acidimicrobiia*	1764	5435	2897	0
		Hyphomicrobium	3085	1550	571	352
		Polaromonas	39	5957	3925	45
		MND1	0	2	0	1
		IS-44	783	3219	2166	3
		Nitrospira	11808	13207	9935	893
EC:1.8.1.2	sulfite reductase (NADPH) hemoprotein	wb1-P19	47872	17264	5766	299
		Uncultured, *Beijerinckiaceae*	32182	20354	10684	298
		Acidobacteria Subdivision 2	943	4429	3696	1
		PLTA13	14336	3557	343	10
		Steroidobacter	1304	296	53	6855
		Ga0077536	549	41	19	6652
		Uncultured, *Burkholderiaceae*	9194	5203	2239	2
		Hyphomicrobium	7686	7199	3388	418
		Polaromonas	20	1108	895	0

		MND1	6	24	14	2
		IS-44	56	10	0	3
		Nitrospira	6	0	0	0
EC:1.8.99.5	dissimilatory sulfite reductase	Ga0077536	142844	57242	18244	97734

Supplemental Table 3. Selection of enzymes related to CO_2 fixation. The EC number abundance predicted by PICRUSt2 show how each dominant taxon is contributing to each predicted enzyme per sample.

EC Number	Enzyme description	Taxa	Abundance			
			**MZ03-3C**	**MZ03-8H**	**MZ03-2B**	**MZ03-10J**
EC 4.1.1.39	Ribulose-bisphosphate carboxylase	Ga0077536	357110	143105	45610	244335
		Uncultured, *Burkholderiaceae*	9194	5203	2239	2
		PLTA13	13472	2832	158	16
		Uncultured, *Beijerinckiaceae*	32182	20354	10684	298
		Polaromonas	18	1955	3759	29
		MND1	1353	2424	2781	63
		IS-44	444	1457	2243	332

		Hyphomicrobium	491	1330	1975	485
EC:2.7.1.19	Phosphoribulokinase	Ga0077536	71422	28621	9122	48867
		Uncultured, *Beijerinckiaceae*	16091	10177	5342	149
		PLTA13	6736	1416	79	8
		Polaromonas	1	124	264	2
		MND1	892	1567	1350	51
		IS-44	356	516	789	119
		Hyphomicrobium	1686	785	383	616
EC:2.3.3.8	ATP citrate (pro-S)-lyase	*Nitrospira*	26194	27590	21254	1962
EC:1.3.5.4	fumarate reductase (quinol)	All dominant taxa	584664	336100	168909	229927
EC:1.2.7.3/ EC:1.2.7.11	2-oxoglutarate/2-oxoacid ferredoxin oxidoreductase	*Acidobacteria* Subdivision 2	17486	2626	10900	764
		Ga0077536	142844	57242	18244	97734
		Uncultured, *Euzebyaceae*	19134	4064	14	0
		11-24 (*Blastocatellia*)	19659	10329	4116	546
		Crossiella	65601	25915	10409	2899
		Candidatus Methylomirabilis	8001	44877	31128	36

		Elev-16S-976 (*Actinobacteria*)	3248	6021	6729	37
		Uncultured, *Gemmatimonadaceae*	2544	9034	8058	4
		Nitrospira	176	306	388	100
EC:1.2.7.1	pyruvate:ferredoxin oxidoreductase	Ga0077536	142844	57242	18244	97734
		Crossiella	16387	6476	2602	0
		Uncultured, *Euzebyaceae*	9567	2032	7	0
		Uncultured, *Burkholderiaceae*	9194	5203	2239	2
		11-24 (*Blastocatellia*)	6553	3443	1372	182
		Candidatus Methylomirabilis	5334	29918	20752	24
		wb1-p19	1630	11126	7866	40
		Acidobacteria Subdivision 2	943	4429	3696	1
		Elev-16S-976 (*Actinobacteria*)	812	1505	1682	9
		Nitrospira	123871	132451	98542	8650
EC:6.3.4.14	biotin carboxylase	All dominant taxa	264401	168185	86294	63071
EC:6.2.1.1	acetyl-CoA synthase	All dominant taxa	256576	171858	88490	64312

EC:6.4.1.2	Acetyl-CoA carboxylase	All dominant taxa	718207	441981	224296	188801
EC:6.4.1.3	Propionyl-CoA carboxylase	Uncultured, *Beijerinckiaceae*	32182	20354	10684	298
		Uncultured, *Euzebyaceae*	38268	8128	28	0
		Crossiella	114815	45354	18216	5798
		11-24 (*Blastocatellia*)	6553	3443	1372	182
		IMCC26256	2236	15026	8724	0
		Elev-16S-976 (*Actinobacteria*)	5684	10537	11776	65
		Uncultured, *Gemmatimonadaceae*	1272	4517	4029	2
		Polaromonas	78	11914	7850	90
		MND1	70	386	322	9
		IS-44	11	11	103	2
		Hyphomicrobium	9465	8215	4259	3036
EC:4.1.1.31	Phosphoenolpyruvate carboxylase	Ga0077536	71845	28648	9136	52676
		wb1-p19	47872	17264	5766	299
		Crossiella	16414	6481	2602	1449
		Uncultured, *Beijerinckiaceae*	32182	20354	10684	298

		Uncultured, *Euzebyaceae*	9567	2032	7	0
		Uncultured, *Burkholderiaceae*	9194	5203	2239	2
		PLTA13	14336	3557	343	10
		Uncultured, *Gemmatimonadaceae*	1272	4517	4029	2
		Steroidobacter	1304	296	53	6855
		Elev-16S-976 (*Actinobacteria*)	812	1505	1682	9
		Nitrospira	2	0	0	0
		Polaromonas	39	5959	3929	45
		MND1	5	24	13	1
		IS-44	2369	3441	2166	3
		Hyphomicrobium	2690	3105	1563	571
EC:4.2.1.1	carbonic anhydrase	All dominant taxa, except *Rokubacteria*	387218	220535	105867	112758

Supplemental Table 4. Selection of enzymes related to nitrogen metabolism. The EC number abundance predicted by PICRUSt2 show how each dominant taxon is contributing to each predicted enzyme per sample.

EC Number	Enzyme description	Taxa	Abundance			
			MZ03-3C	MZ03-8H	MZ03-2B	MZ03-10J
EC:1.14.99.39	Ammonia monooxygenase	Uncultured, *Beijerinckiaceae*	16091	10177	5342	149
		Hyphomicrobium	24	8	1	5
		Nitrospira	6	9	5	0
		MND1	12	432	259	1
		IS-44	2437	3783	2690	43
EC:1.18.6.1	Nitrogenase	Ga0077536	214266	85863	27366	146601
		Uncultured, *Beijerinckiaceae*	48273	30531	16026	447
		Crossiella	49160	19429	7807	1
		Elev-16S-976 (*Actinobacteria*)	2436	4516	5047	28
		Hyphomicrobium	120	49	31	9
		MND1	3	13	21	2

		IS-44	0	3	8	0
		Polaromonas	0	3	22.5	0
EC:1.4.1.13-14	Glutamate synthase	All dominant taxa	413828	249537	120549	125466
EC:1.4.1.2-4	Glutamate dehydrogenase	PLTA13	14336	3557	343	10
		Steroidobacter	1304	296	53	1304
		Uncultured, *Euzebyaceae*	9567	2032	7	0
		Crossiella	27	5	0	1449
		Ga0077536	423	27	14	3809
		Hyphomicrobium	29	11	2	3
EC:1.4.7.1	Glutamate synthase (ferredoxin)	Uncultured, *Gemmatimonadaceae*	1272	4517	4029	2
EC:1.7.1.15	Nitrite reductase (NADH)	Uncultured, *Beijerinckiaceae*	16091	10177	5342	149
		Uncultured, *Euzebyaceae*	19134	4064	14	0
		11-24 (*Blastocatellia*)	6553	3443	1372	182
		Crossiella	32827	12963	5205	2899
		Uncultured, *Burkholderiaceae*	18388	10406	4478	4

		Elev-16S-976 (*Actinobacteria*)	1624	3011	3365	19
		Ga0077536	126	14	5	2843
		Hyphomicrobium	1986	735	142	45
EC:1.7.2.1	Nitrite reductase (NO-forming)	Uncultured, *Gemmatimonadaceae*	1272	4517	4029	2
		Hyphomicrobium	1986	735	142	45
EC:1.7.2.4	Nitrous-oxide reductase	Uncultured, *Gemmatimonadaceae*	1272	4517	4029	2
		Hyphomicrobium	1986	735	142	45
EC:1.7.2.5	Nitric-oxide reductase (cytochrome c)	*Hyphomicrobium*	4242	2729	1128	553
		MND1	1	5	11	1
		IS-44	7	7	53	1
		Polaromonas	1	68	159	1
EC:1.7.7.1	Ferredoxin--nitrite reductase	Uncultured, *Beijerinckiaceae*	16091	10177	5342	149
		Hyphomicrobium	2729	2162	937	74
EC:1.7.99.1	Hydroxylamine reductase	Ga0077536	71422	28621	9122	48867
		MND1	2	2	14	1

		IS-44	0	3	3	0
		Nitrospira	438	486	716	69
		Hyphomicrobium	4	0	0	0
		Polaromonas	0	0	8	0
EC:1.7.99.4	Nitrate reductase	wb1-p19	47872	17264	5766	299
		Uncultured, *Beijerinckiaceae*	16091	10177	5342	149
		PLTA13	6736	1416	79	8
		Uncultured, *Euzebyaceae*	9567	2032	7	0
		Hyphomicrobium	5188	5200	2467	647
		Uncultured, *Burkholderiaceae*	9194	5203	2239	2
		Polaromonas	156	23823	15703	96
		MND1	0	1	1	2
		IS-44	1	1	0	0
		Nitrospira	39398	41455	31008	3351
		11-24 (*Blastocatellia*)	13106	6886	2744	364

EC:4.2.1.1	Carbonic anhydrase	All dominant taxa, except *Rokubacteria*	387218	220535	105867	112758
EC:2.7.2.2	Carbamate kinase	*Polaromonas*	0	5	7	0
		MND1	0	0	1	0
		Nitrospira	1	11	14	0
		Hyphomicrobium	1	1	2	0
EC:3.5.1.49	Formamidase	*Hyphomicrobium*	15	7	7	0
		Polaromonas	48	6788	5218	26
		MND1	0	0	6	0
		IS-44	0	2	42	1
EC:3.5.1.5	Urease	PLTA13	20208	4248	237	24
		Ga0077536	214266	85863	27366	146601
		Uncultured, *Euzebyaceae*	9567	2032	7	0
		Crossiella	49187	19434	7807	1450
		Uncultured, *Burkholderiaceae*	27582	15609	6717	6
		Polaromonas	132	18717	12801	144

		Elev-16S-976 (*Actinobacteria*)	2436	4516	5047	28
		Hyphomicrobium	560	3217	2102	1579
		Nitrospira	4243	2661	2115	258
		IS-44	0	16	55	0
		MND1	0	2	2	2
EC:4.2.1.104	Cyanase	*Hyphomicrobium*	16	6	1	1
		Nitrospira	12972	13623	10353	906
		Polaromonas	45	6334	4481	62
		MND1	0	0	4	0
EC:6.3.1.2	Glutamate-ammonia ligase	All dominant taxa	333052	240385	126438	68775
